# Nanoscale Heterogeneity of Multilayered Si Anodes with Embedded Nanoparticle Scaffolds for Li‐Ion Batteries

**DOI:** 10.1002/advs.201700180

**Published:** 2017-08-08

**Authors:** Marta Haro, Vidyadhar Singh, Stephan Steinhauer, Evropi Toulkeridou, Panagiotis Grammatikopoulos, Mukhles Sowwan

**Affiliations:** ^1^ Nanoparticles by Design Unit Okinawa Institute of Science and Technology (OIST) Graduate University 1919‐1 Tancha Onna‐son Okinawa 904‐0495 Japan

**Keywords:** cluster beam deposition, hybrid materials, Li‐ion batteries, nanoparticles, Si anodes

## Abstract

A new approach on the synthesis of Si anodes for Li‐ion batteries is reported, combining advantages of both nanoparticulated and continuous Si films. A multilayered configuration prototype is proposed, comprising amorphous Si arranged in nanostructured, mechanically heterogeneous films, interspersed with Ta nanoparticle scaffolds. Particular structural features such as increased surface roughness, nanogranularity, and porosity are dictated by the nanoparticle scaffolds, boosting the lithiation process due to fast Li diffusion and low electrode polarization. Consequently, a remarkable charge/discharge speed is reached with the proposed anode, in the order of minutes (1200 mAh g^−1^ at 10 C). Moreover, nanomechanical heterogeneity self‐limits the capacity at intermediate charge/discharge rates; as a consequence, exceptional cycleability is observed at 0.5 C, with 100% retention over 200 cycles with 700 mAh g^−1^. Higher capacity can be obtained when the first cycles are performed at 0.2 C, due to the formation of microislands, which facilitate the swelling of the active Si. This study indicates a method to tune the mechanical, morphological, and electrochemical properties of Si electrodes via engineering nanoparticle scaffolds, paving the way for a novel design of nanostructured Si electrodes for high‐performance energy storage devices.

## 1 Introduction

Since their commercialization in 1991, Li‐ion batteries (LIBs) have dominated the portable electronics market, prevailing over alternative energy storage media.^1^ Nevertheless, more breakthroughs are necessary for the realization of large‐format LIBs, essential for the electric vehicle industry and for energy storage from intermittent renewables in grids, or of miniaturized LIBs for autonomous microelectronics devices like microscale wireless sensors, portable and implantable medical devices, or smart textiles. Despite each of these applications having specific requirements, some aims such as the simultaneous increase of energy and power density are common in order to optimize battery operation.

Si‐based materials are promising anode candidates for LIBs due to the high theoretical capacity (e.g., 4200 mAh g^−1^ as Li_22_Si_5_ or 3579 mAh g^−1^ as Li_15_Si_4_) and low discharge potential (≈0.5 V vs Li/Li^+^) of Si.^2^ However, associated with large capacity is extensive volumetric expansion (300–400%) that results in the accumulation of mechanical stresses causing fractures in the electrode with the resultant loss of active material and electrical conductance.^3^ Other drawbacks for Si anode implementation are the low Li diffusion coefficient (10^−13^–10^−12^ cm^2^ s^−1^)^4^ that restricts the usage of full capacity and rate performance. Alternatively, a recent study^5^ shows a high rate performance of Si anodes during the delithiation process (72% of the total available capacity in 22 s) but points at the lithiation as the limiting process.

In an attempt to suppress mechanical damage and boost high rate capability, extensive research has been performed on the fabrication of nanostructured Si electrodes in the form of nanotubes,^6^ nanowires,^7^ 3D Si membranes,^8^ or hollow spheres;^9^ however, regrettably, the use of nanostructured Si also proved to increase the liquid electrolyte degradation that causes premature failure of the electrode.^10^ The design of composite structures has been suggested to help avoid this effect: various core–shell structures,^11^ nanowire and nanotube heterostructures,^12^ Si nanoparticles (NPs) embedded in conductive matrices,^13^ or, most recently, multilayered structures with graphene were proposed to inhibit side reactions between the nanostructured Si and the electrolyte.^14^ Unfortunately, the synthesis of nanostructured Si can be challenging;^15^ furthermore, it usually needs the employment of solvents, which can limit the application potential of LIBs.

Physical vapor deposition (PVD) of Si thin films, on the other hand, does not require the use of solvents, additives, or binders; offers low‐resistance and high‐purity films; and is a suitable technique for both fundamental research and miniaturized devices such as smart cards, sensors, and microelectromechanical systems.^16^ Nevertheless, the direct use of PVD‐grown Si films suffers from thickness limitations (≈100 nm), above which the electrode is pulverized after a few cycles due to mechanical stress inside the film,^17^ and limited charge–discharge (C–D) response rates.^5^ As a result, it would be desirable to combine nanostructural characteristics and PVD Si thin films in order to bypass these problems, with the resultant improvement of their performance in LIBs.

Herein, we propose a new approach to synthesize nanostructured electrodes based on the use of NP scaffolds interlayered within amorphous Si (a‐Si) layers in order to exploit the advantages of both nanoparticulated and continuous Si films without suffering from their respective shortcomings. The current prototype is fully synthesized by PVD, comprising an a‐Si layer deposited by radio frequency (RF) sputtering and Ta NP scaffolds fabricated by cluster beam deposition (CBD). This prototype anode benefits from being synthesized directly on the substrate (making it easy to incorporate to any microelectromechanical device) without the addition of any binder or chemical solvent, allowing versatile, one‐pot synthesis with excellent control of thickness and size of the deposited material.^18^ Metallic Ta is selected as the NP scaffold material because of its chemical stability (not reacting with either Li or Si), excellent suitability for NP synthesis combined with size selection, and postgrowth behavior (e.g., oxidation and sintering).^19^ These properties allow an in‐depth evaluation of the NP scaffold's effect on the structure, morphology, and nanomechanical properties of the a‐Si layers, and of the electrochemical performance of the synthesized anode.

Sequential deposition of alternating Ta NP and a‐Si layers provides an anode with a multilayered configuration (henceforth, called Si/Ta ML anode), where a‐Si assembles in mechanically heterogeneous, porous structures onto Ta NP scaffolds, as demonstrated by materials' characterization using scanning electron microscopy (SEM), atomic force microscopy (AFM), X‐ray photoelectron spectroscopy (XPS), and peak force quantitative nanomechanical (PF‐QNM) property). This unique nanostructure leads to a markedly different electrochemical behavior compared with a‐Si reference anodes in terms of kinetics, capacity, and cycleability. The Si/Ta ML anode delivers fast C–D cycleability (1200 mAh g^−1^ at 10 C and 200 mAh g^−1^ at 100 C) due to fast Li diffusion that causes low polarization of the electrode, while the a‐Si reference anode shows no signs of the lithiation process at a C–D rate of 10 C or above. In spite of the increased porosity of the Si/Ta ML anode, self‐limiting capacity is observed when the anode is cycled at 0.5 C, providing an excellent capacity retention of 100% along 200 cycles, with a value of 700 mAh g^−1^. This effect is explained by the nanomechanical heterogeneity of the Si/Ta ML anode. This study shows that through engineering underlayers of porous NP scaffolds, it is possible to manipulate the morphological, mechanical, and electrochemical properties of the a‐Si layers, providing a versatile synthesis approach with promising perspectives in the design of nanostructured Si anodes. The prototype, presented here as a fundamental study, offers great potential for the optimization of nanostructured Si anodes for feasible practical applications, either by direct upscaling (utilizing PVD or other techniques) or by integration in microbatteries (since it can be directly deposited on any chip without any chemical contamination and adapted to any 3D structure).

## 2 Results and Discussion

### 2.1 Synthesis and Characterization of Si/Ta ML Anode

**Figure 1:**
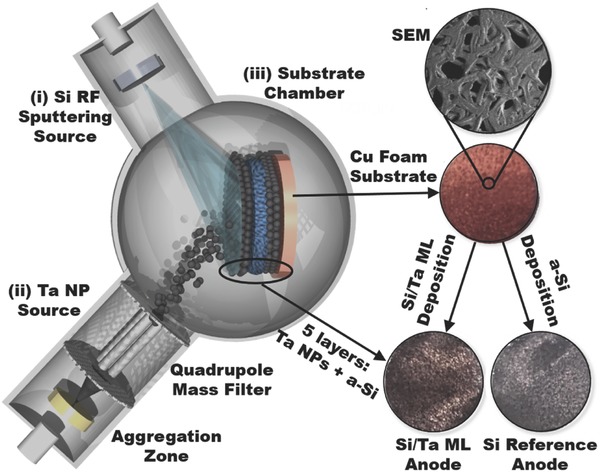
Schematic representation of the magnetron‐sputtering gas‐phase condensation setup used to prepare the Si/Ta ML anode: (i) the Si RF sputtering source, (ii) the Ta nanoparticle (NP) source comprising the aggregation zone and the quadrupole mass filter, and (iii) the substrate chamber at high vacuum. Photographs of the Cu foam substrate (as well as an SEM magnification) and the Si/Ta ML and Si reference anodes are shown on the right.

For the fabrication of Si/Ta ML anodes, the selected substrate was Cu foam because of its 3D macroporous structure, which is rough and flexible, offers large electrode/electrolyte interface, and alleviates environmental stress to partly accommodate volume expansion.^20^ **Figure** **1** shows a photograph of the Cu foam substrate and details of the 3D structure obtained by SEM. The Cu foam substrate was held in the substrate chamber of the gas‐phase deposition system (details in ref. 21) as schematically illustrated in the left‐hand side of Figure 1. Once a high vacuum was reached, synthesis of the Si/Ta ML anode was carried out by five sequential depositions of Ta NP films by the CBD source and Si thin films by the radio frequency magnetron sputtering (RF‐MS) source. A photograph of the resultant Si/Ta ML anode is also shown in Figure 1, along with a photograph of an Si reference anode synthesized under the same conditions as the Si/Ta ML anode. A marked color change can be observed between the Si/Ta ML and Si anodes due to the Ta NP scaffolds being intercalated between the Si layers. Once the samples were synthesized, they were directly transferred to a glove box by a load‐lock mechanism, thus avoiding any environmental or O_2_ contamination.

**Figure 2:**
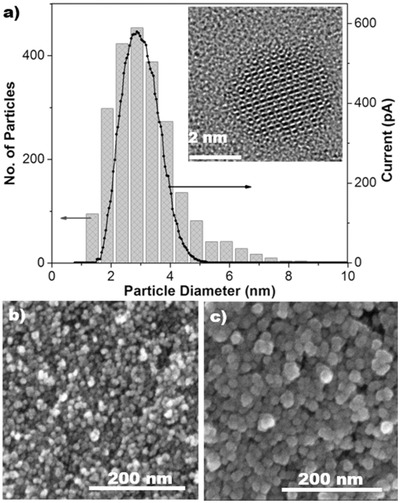
a) Ta nanoparticle size distribution histogram determined by AFM (Figure S1a, Supporting Information) and quadrupole mass filter (QMF) size selection profile (solid line). Inset: HRTEM of Ta NP embedded in a‐Si. SEM images of Ta NPs scaffold on Cu foam, covered by b) 3 nm, and c) 15 nm of a‐Si thin film.

Next, we decompose the Si/Ta ML anode in its constituents in order to scrutinize its features. The lowermost layer is a Ta NP scaffold, ≈10–15 nm in thickness,[[qv: 19a]] which prevents the delamination of Si from the Cu substrate. This delamination has been ascribed to diffusion of Cu atoms inside Si,^22^ frictionless sliding between Si and Cu caused by Li segregation at the Li*_x_*Si/Cu interface,^23^ or stress differences between Si and Cu surfaces.^24^ In order to avoid coalescence phenomena and to ensure a porous scaffold, the Ta NPs were deposited without applying any bias potential, with their kinetic energy of landing mainly governed by the pressure differential between the aggregation zone and the deposition chamber (≈2.3 × 10^−4^ mbar during sputtering). Under these conditions, soft landing can be assumed, with a landing energy lower than 0.1 eV per atom.^25^ **Figure** **2**a shows a narrow size distribution of Ta NPs centered at 3 nm, as determined by AFM and corroborated by TEM (Figure S1, Supporting Information), in good agreement with the reading of the quadrupole mass filter used. The Ta NPs have a tetragonal (β phase) crystalline structure, as observed by high resolution TEM (HRTEM) (inset, Figure 2a), similar to Ta thin films previously reported in literature.^26^

Subsequently, an Si thin film (≈35 nm) was deposited by the RF‐MS source, at a rate controlled with a quartz crystal thickness monitor. In order to evaluate the growth of a‐Si on the Ta NP scaffolds, two samples were synthesized: (i) one layer of Ta NPs covered by ≈3 nm of Si (Figure 2b) and (ii) one layer of Ta NPs with ≈15 nm Si layer (Figure 2c). Both figures show a radial growth of Si coating the Ta NPs. As result, a porous Si layer is formed, with plenty of cavities on top of the Ta NP scaffold. With increased Si sputtering time, the grains became larger, and the porosity decreased; nevertheless, most importantly, the a‐Si layer maintained its nanostructure.

**Figure 3:**
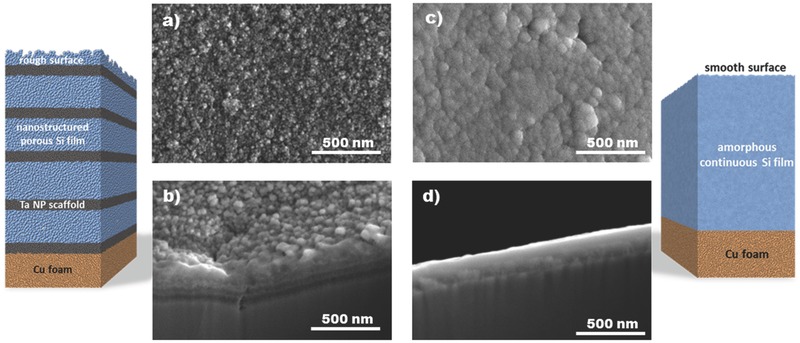
Schematic representations of an Si/Ta ML anode (left) and an Si anode (right). The Si/Ta ML anode scheme shows two phases: nanostructured porous Si film and Ta NP scaffolds, consisting of Ta NPs and Si (qualitatively estimated Si/Ta ratio of 70/30 at% by XPS in Figure S2a, Supporting Information). Surface and cross‐sectional SEM images of a,b) Si/Ta ML anode and c,d) Si anode. The sample thicknesses are ≈200 and 160 nm for Si/Ta ML and Si anodes, respectively, which were evaluated from the cross‐sectional SEM images.

The deposition of Ta NP scaffolds and a‐Si layers was repeated five times to achieve an ML structure, as schematically illustrated in **Figure** **3** (left); note that the topmost Si layer is thinner than the others (10 nm) to increase the electrode–electrolyte interface. As the representative SEM image of Figure 3a confirms, the surface of the Si/Ta ML anode is rough and nanostructured, with granules being a few nanometers in size due to the underlying Ta NP scaffold. The ML structure is clearly demonstrated in Figure 3b, where alternating layers of dissimilar brightness are discerned, indicating the separation of two phases. The atomic compositions of these two phases were studied by XPS with controlled Ar^+^ etching (Figure S2a, Supporting Information), showing the evolution from a practically pure‐Si phase to a rich‐Ta phase. This result highlights the presence of Si atoms distributed all along the Si/Ta ML anode, simultaneously confirming the porosity of the Ta NP scaffolds. This experimental result agrees with the estimated volume occupied by Ta in the NP scaffold, which is ≈20% (see the Supporting Information for details). Furthermore, XPS data show that Ta NPs are in the metallic state, with negligible—if any—formation of Ta silicides at the Ta/Si interface (Figure S2b,c, Supporting Information). In contrast to the Si/Ta ML anode, when Si is deposited on Cu foam in the absence of Ta NP scaffolds, a comparatively smooth and homogeneous film with large‐scale features is obtained, as shown in Figure 3c,d. A schematic representation of an Si reference anode is depicted in Figure 3 (right). From the SEM cross‐sectional images (Figure 3b,d) it can be estimated that the thickness of the Si/Ta ML anode is 40 nm larger than that of the Si anode, a value much higher than the expected 6 nm calculated considering that the atomic percentage of Ta in the Si/Ta ML anode is 3.5 at% and Ta as a solid cylinder (see the Supporting Information for details). Therefore, it can be estimated that 15–20% of the Si/Ta ML anode volume is void space and is correlated to the porosity of the composite electrode. The smoother surface of the a‐Si anode in comparison to the nanostructured Si/Ta ML anode is verified by AFM surface roughness measurements (Figure S3, Supporting Information), showing that root mean square (RMS) roughness increases from 5.82 to 10.83 nm for the Si and Si/Ta ML anodes, respectively.

**Figure 4:**
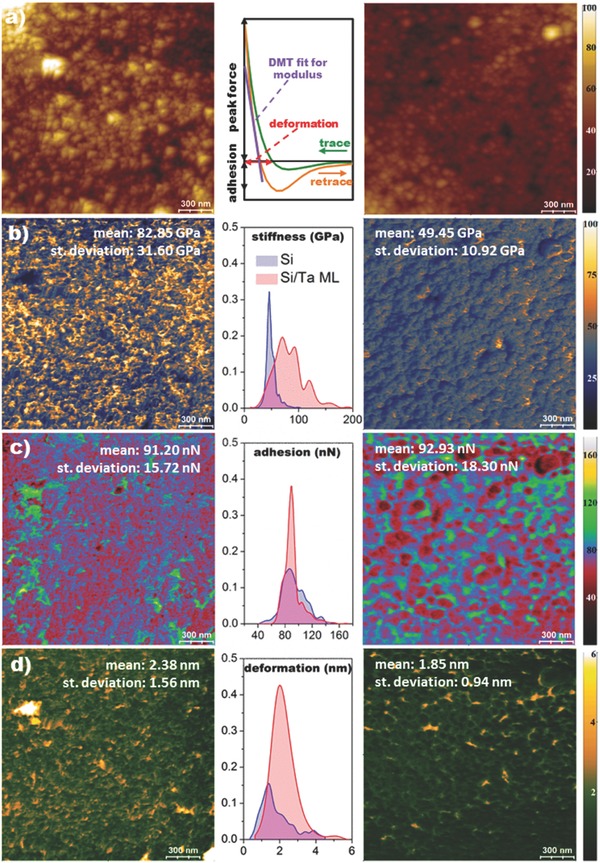
Nanomechanical properties obtained using PF‐QNM mode for Si/Ta ML (left) and a‐Si (right) anodes. a) Topographical AFM images, b) stiffness, c) adhesion, and d) deformation. For each property, the probability density function is shown in the center for both types of anodes. All property values are obtained from force versus separation curves such as that in part (a).

The transition from a homogeneous (Si anode) to a heterogeneous (Si/Ta ML anode) structure with the incorporation of the Ta NP scaffolds is also reflected in the nanomechanical properties. **Figure** **4**a shows AFM topographic images indicating various surface features of the Si/Ta ML and Si anodes, for comparison with corresponding nanomechanical property mappings. Force–distance curves obtained by PF‐QNM at 525 nN provide the stiffness, adhesion, and deformation according to the scheme shown in Figure 4a (middle). The mapping for each mechanical property is shown in Figure 4b–d, together with the calculated probability density function. The Si/Ta ML anode stiffness (Figure 4b) shows a pronounced heterogeneity in nanoregions smaller than the morphological grains observed by AFM. The probability density function of the Si anode stiffness forms a narrow distribution centered at 49 GPa, in contrast to the wider one obtained for the Si/Ta ML anode, which ranges from 20 to 200 GPa, showing a main peak at 83 and a shoulder at 49 GPa—the value obtained for the Si sample. Increase in stiffness of composite films has been ascribed to the effect of NP reinforcement^27^ or the enhancement of the film's granularity.^28^ Here, the lack of direct correspondence with the morphology and the observation of similar adhesion values for the a‐Si and Si/Ta ML anodes (Figure 4c) imply a combination of both. The other effect observed from the nanomechanical study is an increase in the number of voids within the Si matrix induced by the incorporation of Ta NPs, promoting an increase in deformation (Figure 4d). The deformation of the a‐Si anode ranges from 0.5 to 4.5 nm with a mean value of 1.85 nm, while the Si/Ta ML anode shows a mean value of 2.38 nm (≈0.4 of probability density), with deformation values extending up to 12 nm. The formation of voids has been reported for multilayered films incorporating NPs during the deposition.^29^

### 2.2 Electrochemical Performance

**Figure 5:**
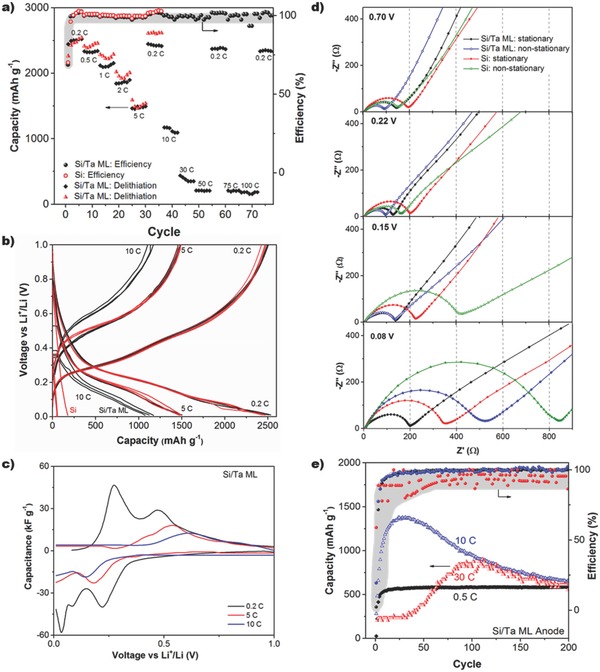
Electrochemical characterization of Si/Ta ML and a‐Si anodes. a) Capacity during delithiation of Si/Ta ML and Si anodes during cycling at different C–D rates and the corresponding Coulombic efficiency. b) C–D plots of Si/Ta ML and Si anodes at 0.2, 5, and 10 C. For 0.2 and 5 C, the cycles shown are from 2 to 4 since they are more representative than the first cycle, while for 10 C the cycles depicted are from 1 to 3 to discuss the first response of Si anode. c) d*Q*/d*V* obtained from the second C–D plot of Si/Ta ML anode. d) Nyquist plot of Si/Ta ML and Si anodes from EIS measurements performed at stationary and nonstationary conditions. e) Capacity of Si/Ta ML anode at 0.5, 10, and 30 C and the Coulombic efficiency along 200 cycles.

Si/Ta ML and Si anodes were assembled in semibatteries, where the reference as well as counter electrode is Li foil, to test the electrochemical performance by C–D plots and electrochemical impedance spectroscopy (EIS). The charge capacity (delithiation) and Coulombic efficiency for the Si/Ta ML and Si anodes are depicted in **Figure** **5**a, showing that, at low and intermediate C–D current (from 0.2 to 5 C), the incorporation of Ta NPs in the Si/Ta ML anode generates no significant change in the capacity with respect to the Si reference anode. As expected, the capacity of the first discharge (lithiation) is higher than the charge, due to the formation of the solid electrolyte interface (SEI), giving a Coulombic efficiency close to 70%, which increases and is maintained at around 100% in the following cycles. At 0.2 C, both anodes (Si/Ta ML and Si) show high capacity (≈2500 mAh g^−1^), which decreases by 40% of the initial value (≈1500 mAh g^−1^) when the C–D rate rises to 5 C. This capacity decrease is close to the reported theoretical loss with the rate increase in an a‐Si thin film.^5^ In this study, it was estimated that, beyond 5 C, the capacity decreases dramatically due to the sluggish diffusion of Li inside the anode during the lithiation process. In the current study, the a‐Si reference anode does not show any capacity above 10 C, while the Si/Ta ML anode does, suggesting that the nanostructure of the Si/Ta ML anode promotes Li diffusion. The Si/Ta ML anode supplies around 1200 mAh g^−1^ at 10 C, and remarkable storage capacity at the fast C–D rate of 30 and 50 C, with values around 400 and 200 mAh g^−1^, respectively, the latter value remaining constant up to 100 C. These values are of the same order as those recently reported for nanostructured Si anodes,[[qv: 6a,13b,c]] confirming that the Si/Ta ML nanostructure is competitive for high‐rate Si‐based anodes. Furthermore, the Si/Ta ML anode shows promising potential as an anode for microbatteries, with energy and power density values of 160 µWh cm^−2^ µm^−1^ and 4800 µW cm^−2^ µm^−1^ (estimated at the 10 C delithiation process and considering the thickness of Si/Ta ML film alone), especially considering that the values reported for microbatteries with porous electrodes are in the order of 100 µWh cm^−2^ µm^−1^ and 1000 µW cm^−2^ µm^−1^.^30^

The C–D plots of both Si/Ta ML and Si anodes at 0.2 and 5 C are depicted in Figure 5b, showing identical features. At 0.2 C, two plateaus are observed at 0.2 and 0.05 V, attributed to the Li alloying with amorphous silicon by the creation of different amorphous phases (Li_7_Si_3_ and Si_13_Si_4_).[[qv: 4b]] At higher current density (5 C), the plateau at 0.2 V is displaced toward lower voltage, while the latter is hardly visible. The plateaus are clearly observed in the derivative (d*Q*/d*V*) as shown in Figure 5c. The same tendency is observed for the Si/Ta ML anode at 10 C. In contrast, the Si anode shows a capacity of 200 mAh g^−1^ during the first lithiation at 10 C, but when the delithiation process starts, no capacity response is measured. Similar behavior is observed in cycles 2 and 3 (with a lithiation capacity of 50 mAh g^−1^).

SEM images of Si/Ta ML and Si anodes recorded after three cycles of charge and discharge at 10 C (Figure S4, Supporting Information) show no pulverization signs or cracks in the Si anode, suggesting kinetic limitations for Si lithiation, while the Si/Ta ML anode maintains a consistent multilayered structure. This result is consistent with the previous reports in which high capacity was registered for a‐Si thin films (50 nm) at high current C–D rates (12 and 30 C) after an activation process of several hundred cycles.^31^

In order to evaluate the difficulty of lithium intake inside the Si and Si/Ta ML anodes at different C–D rates, the charge transfer resistance (*R*_ct_) obtained from EIS spectra^32^ was measured at different voltages: (i) stationary conditions (a voltage approximation of 50 µVs^−1^ after three C–D cycles at 0.2 C) and (ii) nonstationary conditions (a voltage approximation of 150 µVs^−1^ after three C–D cycles at 1 C). Representative Nyquist plots are depicted in Figure 5d, and the values obtained for *R*_ct_ collected in Table S1 (Supporting Information). The arc observed at high frequency of the Nyquist plot is associated with the interfacial phenomena between the electrode and the electrolyte. In Figure 5d, it is observed that the arc changes at potentials approaching alloying reactions, indicating that the mechanisms occurring at the interface are influenced by the state of charge of the electrode.[[qv: 32a]] At voltage above that of the Si lithiation reaction (*V* > 0.22 V), Si/Ta ML and Si anodes show *R*_ct_ values below 200 Ω. At lower voltage (0.15 V), where the lithiation of Si takes place, the *R*_ct_ value of the Si anode increases notably, particularly in nonstationary conditions, while low *R*_ct_ values are maintained for the Si/Ta ML anode. At lower voltage, i.e., 0.08 V, when there is a transition from Li_7_Si_3_ to Si_13_Si_4_, the *R*_ct_ value of the Si anode is practically double that obtained for the Si/Ta ML anode at both stationary and nonstationary conditions. Furthermore, the *R*_ct_ values obtained for nonstationary conditions are larger than at stationary conditions. *R*_ct_ is a measure of the electron transfer from the Si surface to the Li^+^ cation at the electrode/electrolyte interface, and is related to the polarization of the electrode.^33^ Therefore, *R*_ct_ evaluates the kinetic limitation of the Li^+^ reduction at the electrode/electrolyte interface and the absorption inside the Si anode. *R*_ct_ depends on the state of charge of the electrode and on the kinetic experimental conditions (stationary or nonstationary), which suggests that *R*_ct_ is related to the Li atoms at the electrode surface and the Li diffusion from the surface toward the interior of the electrode.^33^ Subsequently, when Li atoms have time to diffuse inside the Si, unoccupied surface Si lattice sites are available to introduce new Li atoms by a charge‐transfer process without additional resistance. Therefore, Figure 5d suggests that Li diffusion in the Si/Ta ML anode is faster than in the Si anode, probably facilitated along the grain boundaries of the porous nanostructure of the Si/Ta ML anode. As a result, the Si/Ta ML anode can provide capacity at fast C–D rate; in contrast, lithiation is kinetically limited in the Si anode by the accumulated Li on the surface of the electrode and no new Li intake occurs.

So far, we have described the fast C–D rate of the Si/Ta ML anode in terms of the nanoporosity and nanostructure of the synthesized anode. Nevertheless, the nanomechanical heterogeneity also plays a critical role on the electrochemistry performance of the Si/Ta ML anode. This effect is observed in Figure 5e, which shows the capacity of the Si/Ta ML anode at 0.5, 10, and 30 C over 200 cycles. In the three C–D rates, the capacity is stabilized around 700 mAh g^−1^ (twice the value of current commercial graphite anodes). The convergence to this value can be explained considering the aforementioned nanomechanical properties, which can self‐limit the capacity of Si‐based anodes.^34^ The Si/Ta ML anode shows heterogeneous nanoregions of variable stiffness, some with values similar to the a‐Si anode and others with higher stiffness (double or above), in which Si volume expansion is constrained, limiting the capacity. This effect is clearly observed when the Si/Ta ML anode is cycled at 0.5 C showing a capacity of ≈700 mAh g^−1^, which, however, is increased to 2500 mAh g^−1^ (as in Figure 5a) when the first two cycles are performed at 0.2 C (Figure S5a, Supporting Information). At this low C–D rate, all Si active materials can swell (providing the same capacity as the a‐Si anode), and the resultant stress cracks the Si/Ta ML anode, forming microislands (Figure S5b, Supporting Information); this allows obtaining high‐capacity values but low stability. In contrast, when the cycling is performed at 0.5 C, the constrained volume extension self‐limits the capacity, and the Si/Ta ML anode shows markedly different deformation behavior; as a result, the C–D is reversible, and a capacity retention of 100% is obtained over 200 cycles. At 10 C, the capacity increases during the first cycles until reaching a value of ≈1300 mAh g^−1^, and later decreases until stabilization at ≈700 mAh g^−1^. This “activation” phenomenon has been attributed to two different causes: the enhancement of Li diffusion along fresh Si particle surfaces (i.e., not exposed to the electrolyte) due to a nanocracking effect caused during cycling, and to the reversible decomposition and formation of SEI contributing to the high experimental capacity.^35^ The former explanation is plausible at 10 C, since the few‐minute period during the Si/Ta ML anode lithiation is probably not enough to form a uniform and stable SEI in the first cycles, although the formation of nanocracking cannot be discarded. At even faster C–D rate, such as 30 C, it is necessary to perform the first cycle at 0.2 C to be able to operate the anode along 200 cycles; without this first cycle, only few cycles could be performed. In this case, the first cycle seems to be related to the need for the formation of a stable SEI that cannot be formed at 30 C. The subsequent “activation” after 30 cycles is probably related to nanocracking of the anode.

Self‐limiting capacity such as that observed in the Si/Ta ML anode can be advantageous in applications where long‐lasting batteries are required or in microbatteries where the electrode expansion should be controlled.^36^ Limiting the capacity in Si anodes is a strategy to increase its cycleability by means of limiting the down voltage of lithiation.^37^ Moreover, it is important to highlight that most of the cathodes (except sulfur and air that are under development) are based on the intercalation mechanism, where the capacity is lower than the 700 mAh g^−1^ as provided by our Si/Ta ML anode. Therefore, the Si/Ta ML structure is a promising anode in which the self‐limiting capacity can be tailored by engineering NP scaffolds to control the nanomechanical properties of the electrode in order to customize the electrochemical response.

### 2.3 Effect of Ta NP Scaffold on Lithiation Mechanism

To date, the lithiation mechanism in Si‐based anodes is reported in literature based on three consecutive processes: (i) a redox reaction at the electrolyte/electrode interface (Li^+^ + 1e^−^ → Li), (ii) diffusion of Li through amorphous lithiated silicon (a‐Li*_x_*Si), and (iii) a chemical reaction at the a‐Li*_x_*Si/Si phase boundary^38^ until the a‐Si is consumed (with *x* ≈ 2.5); subsequently, a second lithiation process occurs in a single phase, until the final product is obtained (a‐Li*_x_*Si with *x* ≈ 3.75). From the three processes, the second one is the kinetically limiting step, due to the low Li diffusion coefficient (10^−14^–10^−13^ cm^2^ s^−1^)^4^ registered for Si, which restricts the lithiation rate of Si anodes.^5^ Also the large ratio of Li:Si atoms (≈3.75:1) in the lithiated state of a‐Li*_x_*Si introduces great stress inside the Si anode, which produces the crack of the electrode and the loss of contact between the Si and the substrate, reducing the capacity during C–D cycling.

**Figure 6:**
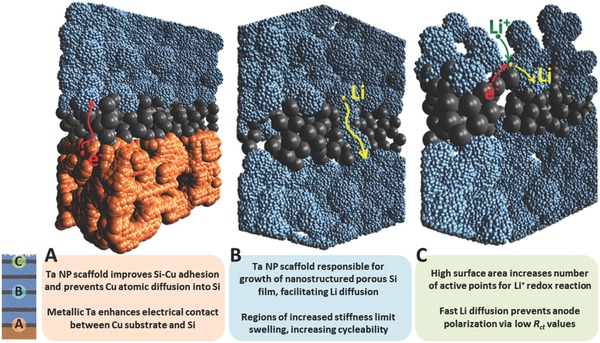
Schematic representation of Si/Ta ML anode with three magnified areas: A—Ta NP scaffold bottom layer; B—representative Ta NP scaffold for intermediate layers; and C—Ta NP scaffold of the top layer. These illustrations show the main effects of the Ta NP scaffolds on the nanostructure and nanomechanical properties of the a‐Si anode material, and the resulting influence on the electrochemical performance of the Si/Ta ML anode during the lithiation process.

Based on this, the Si/Ta ML anode is designed to boost the lithiation process and to provide high capacity retention along cycling. At this point, the design of the Si/Ta ML anode can be evaluated and discussed considering the role of the Ta NP scaffolds on the final morphology and nanomechanical properties of the anode, and the electrochemical performance when assembled in an Li‐ion battery. For this purpose, **Figure** **6** shows schematic illustrations of three different layers of the Si/Ta ML anode which provide important insights into the effect of the morphology and nanomechanical properties on the lithiation process of the anode.

The first layer grown during the synthesis of the Si/Ta ML anode is the Ta NP scaffold, an interfacial layer between the Cu substrate and the Si active material (illustration A in Figure 6). The NP scaffold provides free space to relieve the stress between the Si active material and the Cu surface, which is one of the main causes of Si delamination from the Cu substrate.^24^ Another reported cause for Si delamination on Cu is the diffusion of Cu atoms inside the Si,^22^ which can also be prevented by the Ta scaffold; note that Ta is extensively used as a diffusion barrier in Si electronics.^39^ Moreover, the Ta NP scaffolds also provide the framework for the nanostructured growth of the Si active material (Figure 2b,c), whereas the high electrical conductivity of Ta favors the electric interfacial contact between the Cu substrate and the Si active material.

Since the size of the Si grains increases with deposition time (Figure 2b,c), intermediate layers of Ta NP scaffolds are intercalated within the Si/Ta ML anode to ensure high porosity, as illustration B in Figure 6 shows. As a result, an ML structure is observed (Figure 3b) with layers of practically pure Si and layers with mixed Si:Ta (Figure S2a, Supporting Information). The formation of Si channels and the high porosity of the anode facilitate fast Li diffusion along grain boundaries in the porous structure. The latter is reflected in the higher deformation values obtained by PF‐QNM (Figure 4d), whereas the presence of intercalated Ta NP scaffolds is also responsible for the increase in stiffness of the Si/Ta ML anode with respect the Si reference anode (Figure 3b). Consequently, self‐limiting capacity is observed at intermediate C–D rate (0.5 C) maintaining 100% capacity retention after 200 cycles (Figure 5e) and providing 700 mAh g^−1^.

The topmost Ta NP scaffold (illustration C in Figure 6) results in a high surface area (Figure 3a; Figure S3a, Supporting Information) that facilitates the redox reaction at the electrolyte/electrode interface (Li^+^ + 1e^−^ → Li), i.e., the first step of the lithiation process, as indicated above. Simultaneously, the high porosity of the top Si layer (as well as that of the ones below) enables fast Li diffusion, controlling the second and the kinetic limiting step of the aforementioned Si lithiation mechanism. As a consequence, the Li atoms have adequate time to diffuse inside the Si/Ta ML anode at high C–D rate, leaving free sites on the surface for further lithiation intake and corresponding low *R*_ct_ values (Figure 5d), indicating low polarization of the electrode. Therefore, the rate capability of the Si/Ta ML anode is improved compared to the Si reference anode (Figure 5a,b).

## 3 Conclusion

A new synthesis approach is proposed for Si‐based anodes, based on multilayered amorphous Si films with Ta nanoparticle scaffolds, deposited by RF sputtering and cluster beam deposition, respectively. Both deposition methods afford one‐pot synthesis with good control over size, chemical state, and purity. The Ta NP scaffolds control the nanostructure, porosity, and nanomechanical heterogeneity of the Si layers. The Si/Ta ML anode shows high rate performance (in the order of minutes and seconds) and low polarity due to the fast Li diffusion along the Si grains into the porous Si/Ta ML anode and the increase of the electrode/electrolyte interface area. Self‐limited capacity is observed at intermediate and fast C–D rates due to the stiffness heterogeneity with regions where the volume expansion is constrained. The lower volumetric expansion produced inside the Si/Ta ML anode under these experimental conditions allows excellent cycleability. The control of self‐limiting capacity allows customizing the electrochemical performance of the anode.

The role of the Ta NP scaffolds on the anode configuration was scrutinized, giving insights into future designs of advanced Si‐based anodes by tuning various parameters, such as the nature, size, and shape of the NPs, or the number and thickness of the Si layers. Ta has been selected herein as a diffusion barrier and its controllable synthesis by CBD; nevertheless, other materials with different properties can be used, such as lower molecular weight or different activity (or inactivity) to lithiation. The size and shape of the NPs control the nanostructure of the a‐Si, which facilitates the Li diffusion inside the electrode and controls the C–D rate of the anode. Also, they determine the accumulated strain and the nanomechanical heterogeneity of the synthesized anode that directly affects its capacity. The size of the a‐Si nanograins depends on the number and thickness of the Si layers, offering the possibility to optimize the balance between fast Li diffusion and high cycleability, and to maximize the amount of a‐Si active material. The number and thickness of the MLs can also be engineered to control the self‐capacity and the cycleability of the synthesized anode by means of the nanomechanical properties. The large number of parameters that can be modified opens many possibilities for the design of advanced Si anodes for Li‐ion batteries based on NP scaffolds.

## 4 Experimental Section

*Si/Ta and Si Anode Deposition*: Si/Ta ML anodes were prepared using a gas‐phase deposition system (Mantis Deposition Ltd) by sequential deposition of Ta NP films^20^ and a‐Si thin films on Cu foam (0.08 mm thickness, MTI Corporation). Silicon (n‐type; purity: >99.999%; resistivity: <0.001 Ω m) and tantalum (>99.95% purity) magnetron sputtering targets were purchased from Kurt J. Lesker. Once Cu foam was properly cleaned (in acetic acid, later ultrasonicated in isopropanol and dried in N_2_), it was placed inside the substrate chamber in high vacuum (2.0 × 10^−8^ mbar) and supported by a rotatory holder (2 rpm for all depositions) to warrant homogeneous film deposition. For Ta NP films, an Ar flow of 60 standard cubic centimeters per minute, a DC magnetron power of 45 W, and an aggregation zone length of 100 mm were selected. The Si thin film was deposited with a 110 W RF‐sputtering source, using an Ar pressure of 2.1 × 10^−3^ mbar. The Si deposition rate and film thickness were measured using a quartz crystal thickness monitor. The Si anodes used as reference were produced following the same deposition conditions as for the Si/Ta ML anode. All the depositions were performed at ambient temperature (298 K, as measured by the substrate holder thermocouple), and with no external bias applied to the substrate. Finally, all the anodes were annealed at 150 °C for 60 min at an Ar pressure of 8 × 10^−3^ mbar.

*Characterization Techniques*: Ta nanoparticles were characterized using an FEI Titan G2 environmental transmission electron microscope equipped with a spherical aberration image corrector (operation voltage: 300 kV). The cross‐sections and surfaces of Si/Ta ML and Si anodes (and Cu foam) were imaged by means of focused ion beam (FIB) milling combined with SEM using an FEI Helios G3 UC FIB–SEM. PF‐QNM measurements (stiffness, adhesion, and deformation) were performed using a handcrafted natural diamond nanoindenting tip (Bruker DNISP‐MM, *k* = 225 N m^−1^ and *f*_0_ = 70 kHz). From the specifications of the manufacturer, the tip radius was assumed to be 40 nm, with a suitable applied load in the range of a few nanonewtons to micronewtons. The tip calibration was performed during measurement on a fused silica standard sample from Bruker, providing an indentation modulus of around 70 GPa. The applied maximum load was set at 525 nN for all measurements.

*Electrochemical Studies*: Electrochemical characterization was carried out using a two‐electrode Swagelok cell with metallic lithium foil as the reference as well as counter electrode. Ethylene carbonate (EC; >99%), diethyl carbonate (DEC; >99%), and lithium hexafluorophosphate (LiPF_6_; >99.99%) for the electrolyte were purchased from Sigma‐Aldrich, and the lithium foil (thickness, 0.38 mm) and Whatman glass microfiber filter (grade GF/C) as the separators. The electrolyte solution was 1.0 m LiPF_6_ in 50:50 (w/w) mixture of EC:DEC. Charge–discharge measurements were performed using an eight‐channel battery analyzer (0.02–10 mA up to 5 V, MTI Corp.) in the voltage window of 0.01–1 V. For the calculation of charge–discharge rate, 1 C was defined as 3579 mAh g^−1^, and 30 µg of active Si material for both Si and Si/Ta ML anodes (calculated from nine a‐Si anodes to reduce the measurement error, 270 ± 10 µg measured with a Sartorius CPA225D balance). The Gamry‐3000 potentiostat used for the EIS measurements. EIS spectra were obtained at different voltages within 0.01–1 V in the discharge (Si lithiation) direction. The amplitude and frequency range were 10 mV and from 1 MHz to 10 mHz, respectively.

## Conflict of Interest

The authors declare no conflict of interest.

## Supporting information

Supplementary
