# Supplementary material for: Nanoscale Heterogeneity of Multilayered Si Anodes with Embedded Nanoparticle Scaffolds for Li‐Ion Batteries
Source: Adv Sci (Weinh). 2017 Aug 8;4(10):1700180. doi: 10.1002/advs.201700180 (PMC5644243; doi:10.1002/advs.201700180)
Supplement: Supplementary file 1 — Supplementary [file ADVS-4-na-s001.pdf]

Copyright WILEY-VCH Verlag GmbH & Co. KGaA, 69469 Weinheim, Germany, 2017.

ADVANCED SCIENCE
Open Access

Supporting Information

for *Adv. Sci.,* DOI: 10.1002/advs.201700180

Nanoscale Heterogeneity of Multilayered Si Anodes with Embedded Nanoparticle Scaffolds for Li-Ion Batteries

*Marta Haro, Vidyadhar Singh, Stephan Steinhauer, Evropi Toulkeridou, Panagiotis Grammatikopoulos, and Mukhles Sowwan**

WILEY-VCH

Copyright WILEY-VCH Verlag GmbH & Co. KGaA, 69469 Weinheim, Germany, 2016.

Supporting Information

Nanoscale Heterogeneity of Multi-Layered Si Anodes with Embedded Nanoparticle Scaffolds for High-Performance Li-Ion Batteries

*Marta Haro, Vidyadhar Singh, Stephan Steinhauer, Evropi Toulkeridou, Panagiotis Grammatikopoulos, Mukhles Sowwan**

S.1. Ta nanoparticle (NP) characterization

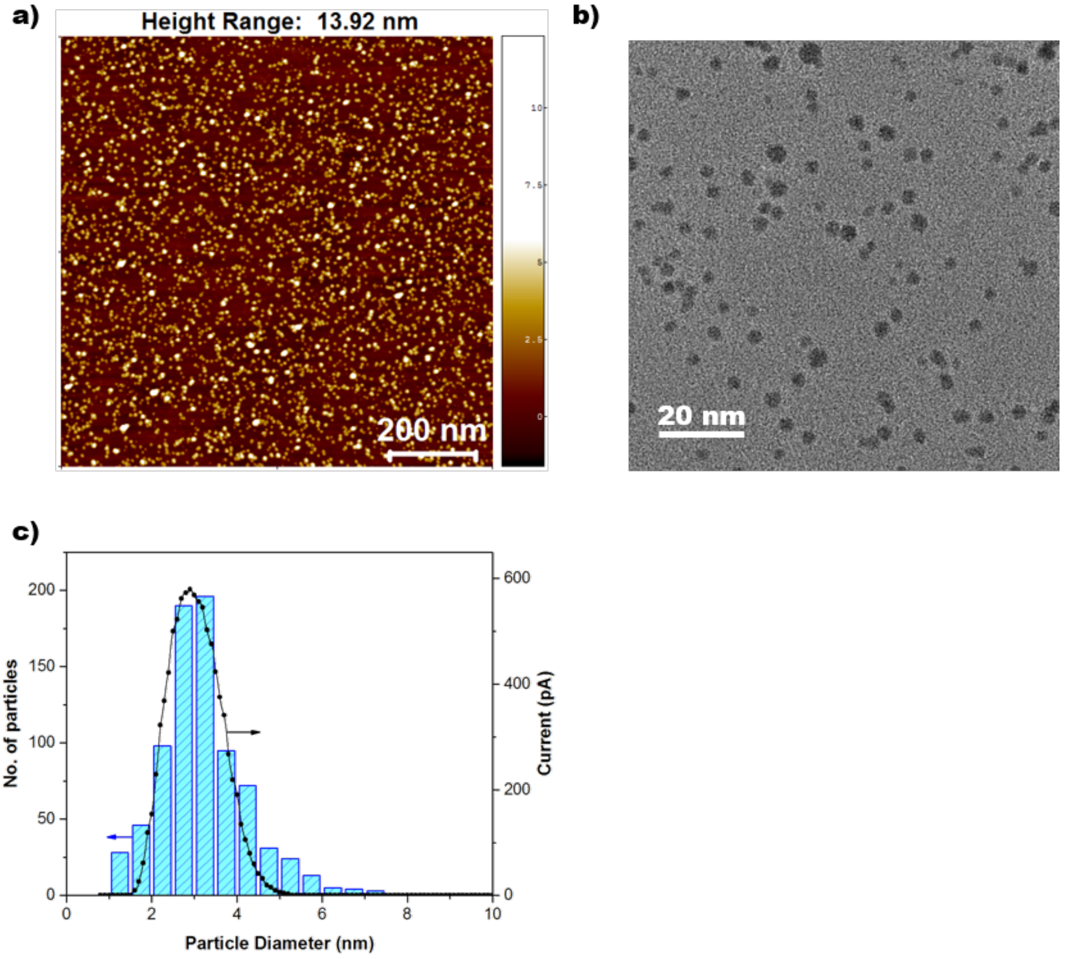

Figure S.1. a) AFM image of Ta NPs deposited on a Si substrate for 5 minutes, used for obtaining the NP size distribution histogram shown in **Figure 2a**. **b)** Exemplary TEM image showing Ta NPs embedded in an amorphous Si matrix. **c)** NP size distribution obtained from 10 TEM images (histogram) and from the quadrupole mass filter (QMF) current (solid line). Good agreement is obtained for the Ta NP size distribution analyzed by AFM, TEM, and the QMF size selection.

Determination of particle size distribution and atomic percentage (at.%), and V of Ta in Si/Ta ML anode

In order to obtain results as close as possible to the fabricated Si/Ta ML anode and to prevent structural changes induced by oxidation, Ta NPs were encapsulated between two amorphous Si thin films (thicknesses of around 7.5 nm and 15 nm for the bottom and top layer,

respectively) on commercial C film TEM grids (Ted Pella). Size distributions were achieved by means of automated image analysis of low-magnification TEM micrographs with the software ImageJ, measuring projected areas of 80 NPs and assuming spherical geometries. Size distributions were analyzed for samples obtained by 5 minutes of deposition, while the Ta NP films in the Si/Ta ML anodes were deposited for 60 minutes, resulting in a porous multilayer structure. From the number of NPs formed within 5 minutes, the volume per particle (~14 nm^3 considering a diameter of 3 nm), the density, and the atomic weights, the Ta/Si ratio was calculated as 3.5 at%. The total volume occupied by Ta in the Si/Ta ML anode can be calculated from the average volume and total number of the Ta NPs within the 5 scaffold layers (~3×10^{13}). A continuous, non-porous, Ta cylinder of this volume and base area of 0.712 cm^2 (i.e. the area of the anode) would have a height of ~6 nm. Since the height difference between the Si/Ta ML and the Si anodes is ~40 nm (from SEM measurements in Figure 3), we can estimate that the void space within the Si/Ta ML anode is around 15-20%. Furthermore, supposing that the 40 nm height difference between the Si/Ta ML and the Si anode corresponds to the 5 NP scaffolds (and can, therefore, be equally divided between them), it can be estimated that the volume occupied by Ta in one NP scaffold is approximately 20%. In other words, the porosity of the Ta NP scaffold is about 80%.

S.2. XPS characterization of Si/Ta ML anode

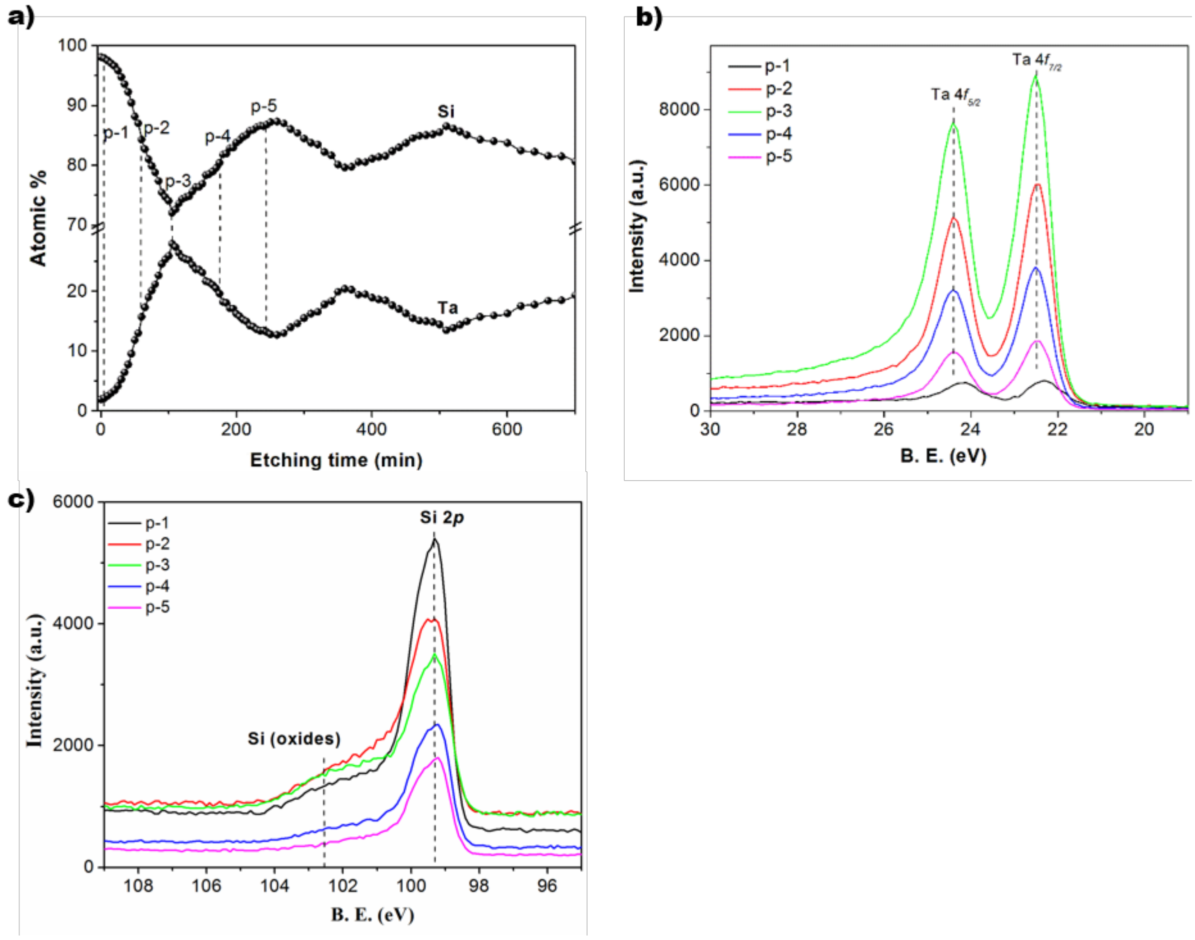

Figure S.2. a) XPS etching profile of the Si/Ta anode showing the atomic distribution of Si and Ta as a function of the etching time (depth), determined by: **b)** Ta *4f* and **b)** Si 2*p* peaks. The accuracy of at.% composition determined by XPS during etching time can be compromised by the 3D structure of the Cu foam. Nevertheless, it clearly shows two distinct layers: almost pure-Si, and Si/Ta with approximately 70% / 30%

$Ta4f_{5/2}$ and $Ta4f_{7/2}$ peaks appear at 24.1 and 22.4 eV, respectively, in the XPS registered in the positions from p-2 to p-5 (shown in Figure SI.2b). These binding energies correspond to Ta in the metallic state,[1] and discard the presence of Ta_2O_5 with binding energies of 29.1 and 27.2 eV for the Ta4f5/2 and Ta4f7/2 peaks. Also Ta silicides can be discarded, since the change from metallic to cationic Ta is associated with a Ta4f peak displacement of 0.6 eV to lower energies.[2] The only position where some Ta silicides could be formed is at p-1, where the T4f peaks appear at 24.1 and 22.3 eV. The Si 2p peak shows that Si oxides exist in the external layer of a-Si (from p-1 to p-3) of the Si/Ta ML anode (the binding energy for SiO_2 is 102.5 eV) that could be formed during the transition from the glovebox to the XPS.

Experimental XPS
XPS measurements were performed in a Kratos AXIS Ultra DLD Photoelectron spectrometer, with an Al Kα (1486.6 eV) source and a base pressure of 10^{-10} mbar. In order to avoid surface contamination, the samples were transferred from the N_2 atmosphere glove box to the XPS chamber using an inert-gas sealed sample holder. The Si 2p, Ta 4f and C 1s core level narrow spectra were recorded using pass energy of 20 eV for high resolution. For depth profiling by means of Ar ion etching, ion energy of 3keV and etch area of around 3 mm × 3 mm were used.

S.3. AFM characterization of Si/Ta ML and Si anodes

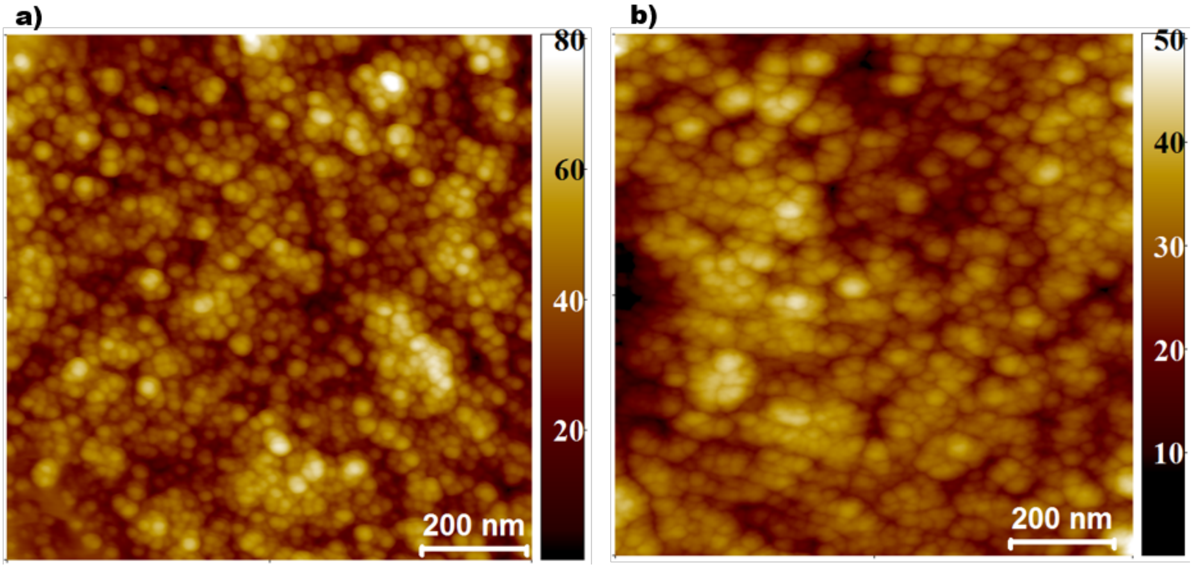

Figure S.3. AFM images of **a)** Si/Ta ML anode, and **b)** Si anode.

AFM characterization
A Bruker Multi Mode 8™ atomic force microscopy (AFM) system equipped with the NanoScope®V controller in tapping mode using triangular silicon-nitride AFM tip (radius <10 nm, force constant 'k' of 0.35 N m^{-1}, 65 kHz of resonant frequency 'f_0'), was used to analyze the surface topography and roughness of the samples. The AFM system height 'Z' resolution and noise floor are less than 0.030 nm.

S.4. SEM characterization of Si/Ta ML and Si anodes after charge-discharge cycling

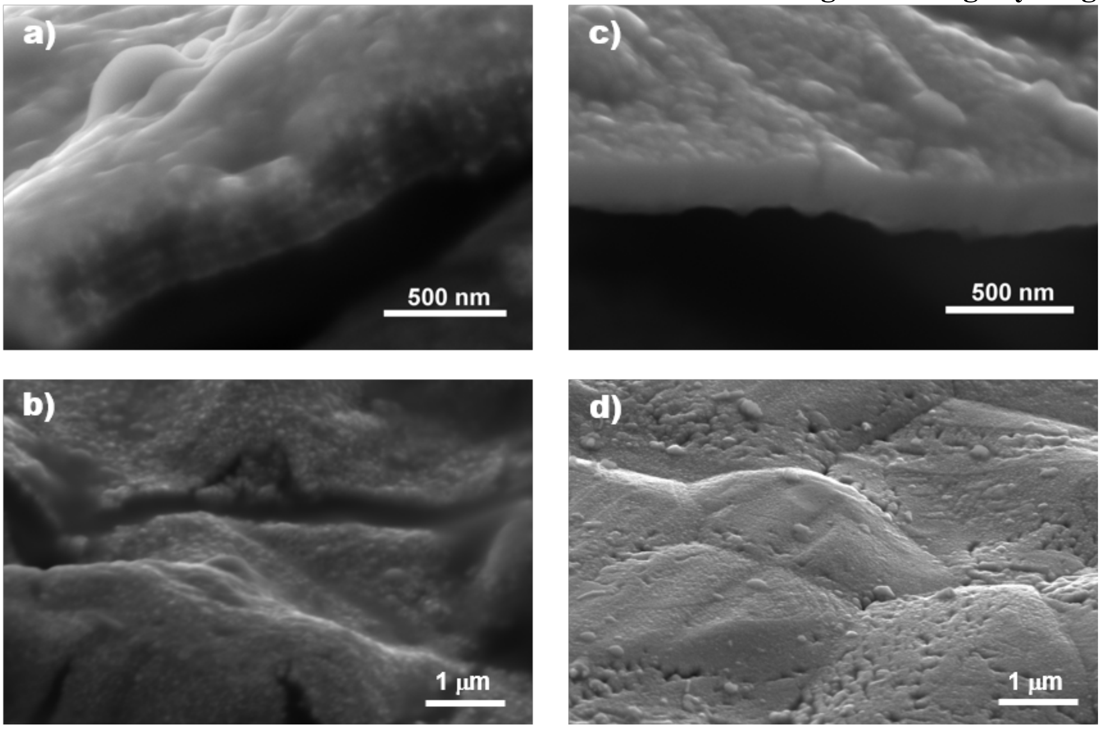

Figure S.4. SEM images of **a), b)** Si/Ta ML and **c), d)** Si anodes after 3 cycles at 10 C.

Experimental SEM characterization after cycling
The semi-batteries were opened inside the glovebox and the anodes cleaned with DEC solvent and isopropanol. After mechanical cutting, the samples were dried in vacuum during 48 hours before SEM characterization. SEM images were obtained with an FEI Quanta 250 FEG.

S. 5. R_{ct} calculation from Nyquist plot

The EIS spectra were recorded at different charge states between 0.01 and 1 V with an arc amplitude of 10 mV in the frequency range from 10^6 Hz down to 10^{-2} Hz. Different treatment was performed for stationary and non-stationary conditions:

- *Stationary conditions*: after 3 cycles of charge and discharge at 0.2 C, the voltage at which the electrochemical impedance spectrum was performed was 50 µV s^{-1}.
- *Non-stationary conditions*: after 3 cycles of charge and discharge at 1 C, the voltage at which the electrochemical impedance spectrum was performed was 150 µV s^{-1}.

The high frequency region of the Nyquist plots are fitted according to the previous equivalent circuit model proposed in ref [3] as two arcs in series formed by a parallel resistance (*R*) and capacitance (*C*). The R of the first arc is associated to the SEI, while the second arc to the R_{ct} and capacitance of the double layer (C_{dl}). The obtained values are shown in Table SI.1. Since the high frequency region is not completely independent on the voltage, the low region frequency region should be also considered in the equivalent circuit model. Nevertheless, the low frequency region is not analyzed because the change of the values is comparatively small, and is not expected to influence the presented conclusions.

Table SI.1. Calculated R_{SEI} and R_{ct} from EIS data for Si/Ta and Si samples.

Conditions	Voltage	Si/Ta ML anode R_{SEI} (Ω)	Si/Ta ML anode R_{ct} (Ω)	Si anode R_{SEI} (Ω)	Si anode R_{ct} (Ω)
Stationary	0.70	14	130	13	179
	0.32	15	118	14	204
	0.22	15	107	14	183
	0.15	17	117	14	206
	0.08	20	174	13	325
	0.01	17	1003	11	537
Non-Stationary	0.70	10	77	10	146
	0.32	10	77	12	126
	0.22	11	77	17	146
	0.15	13	114	17	401
	0.08	10	465	13	788
	0.01	9	498	13	614

It is remarkable that R_{SEI} for Si/Ta ML and Si anodes show similar values, with no increase for the porous Si/Ta ML anode with respect to the Si film. We suggest that the low polarity of the Si/Ta ML anode facilitates the reduction of Li^+ instead of side redox reactions related to the formation of the SEI.

S.6. Complementary results of electrochemical performance of Si/Ta ML anode

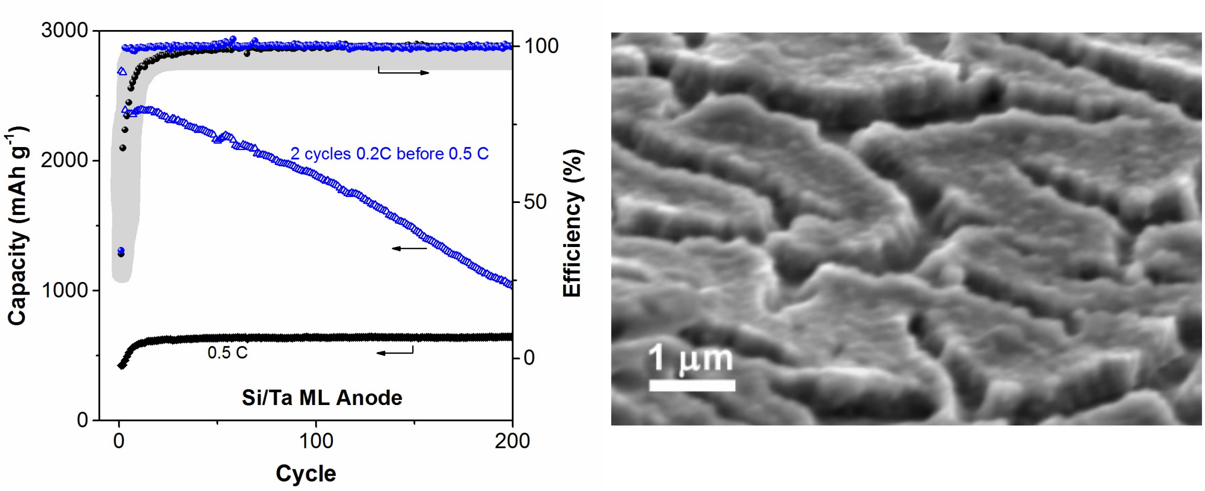

Figure S. 5. Capacity of Si/Ta at 0.5 C registered from different initial conditions (first 2 cycles at 0.2 C and later 0.5 C or directly 0.5 C).

References

[1] E. Atanassova, G. Tyuliev, A. Paskaleva, D. Spassov, K. Kostov, *Appl. Surf. Sci.* **2004**, *225*, 86.

[2] a) M. Zier, S. Oswald, R. Reiche, K. Wetzig, *Anal. Bioanal. Chem.* **2003**, *375*, 902; b) W. Zahn, S. Oswald, A. Fülle, D. Hildebrand, M. Zier, K. Wetzig, *Phys. Status Solidi C* **2007**, *4*, 1830.

[3] M. Haro, T. Song, A. Guerrero, L. Bertoluzzi, J. Bisquert, U. Paik, G. Garcia-Belmonte, *Phys. Chem. Chem. Phys.* **2014**, *16*, 17930.
